# She Always Steps in the Same River: Similarity Among Long-Term Partners in Their Demographic, Physical, and Personality Characteristics

**DOI:** 10.3389/fpsyg.2019.00052

**Published:** 2019-02-05

**Authors:** Zuzana Štěrbová, Petr Tureček, Karel Kleisner

**Affiliations:** ^1^Department of Zoology, Faculty of Science, Charles University, Prague, Czechia; ^2^National Institute of Mental Health, Klecany, Czechia; ^3^Department of Philosophy and History of Science, Faculty of Science, Charles University, Prague, Czechia

**Keywords:** repeatability, intraindividual variability, motherhood, stability of preferences, sexual selection, mating behavior, female preferences

## Abstract

In mate choice, individuals consider a wide pool of potential partners. It has been found that people have certain preferences, but intraindividual stability of mate choice over time remains little explored. We tested individual consistency of mate choice with respect to a number of demographic, physical, and personality characteristics. Only mothers were recruited for this study, because we wanted to find out not only whether women choose long-term partners with certain characteristics but also whether the father of their child(ren) differs from their other long-term (ex-)partners. Women (*N* = 537) of 19–45 years of age indicated the demographic, physical (by using image stimuli), and personality characteristics of all of their long-term partners (partners per respondent: mean = 2.98, *SD* = 1.32). Then we compared the average difference between an individual’s long-term partners with the expected average difference using a permutation test. We also evaluated differences between partners who had children with the participants (fathers) and other long-term partners (non-fathers) using permutation tests and mixed-effect models. Our results revealed that women choose long-term partners consistently with respect to all types of characteristics. Although effect sizes for the individual characteristics were rather weak, maximal cumulative effect size for all characteristics together was high, which suggests that relatively low effect sizes were caused by high variability with low correlations between characteristics, and not by inconsistent mate choice. Furthermore, we found that despite some differences between partners, fathers of participants’ child(ren) do fit their ‘type’. These results suggest that mate choice may be guided by relatively stable but to some degree flexible preferences, which makes mate choice cognitively less demanding and less time-consuming. Further longitudinal studies are needed to confirm this conclusion.

## Introduction

Human mate choices are influenced by various sociodemographic, physical, and psychological characteristics of a prospective partner. Majority of research on absolute partner preferences focuses on what is considered attractive across various individuals (see e.g., Buss, 1989; Regan et al., 2000). This line of research yielded evidence on high agreement with respect to attractiveness both within and across cultures (*r* > 0.90) (see a meta-analysis, Langlois et al., 2000). Agreement in attractiveness assessment between individual raters is, however, much lower (*r* > 0.50) (see a meta-analysis, Feingold, 1992). It seems therefore that despite a strong general consensus in attractiveness assessments in general, there exists a substantial variability between individual preferences (Hönekopp, 2006). This interindividual variability may be due to relative partner preferences (e.g., based on own characteristics and experiences, Figueredo et al., 2006; see a review, Štěrbová and Valentová, 2012). It is also possible, moreover, that an individual’s partner preferences also change over time (Kościński, 2010).

In non-human animals, it has been found that individual consistency of female mate preferences is rather low and context-dependent, because it varies depending on females’ age, environment, and conditions (Cotton et al., 2006; Jennions and Petrie, 2007; Bell et al., 2009). In humans, ontogeny of mate preferences has been studied mostly cross-sectionally (Brumbaugh and Wood, 2013; Boothroyd and Vukovic, 2018). This approach revealed differences among various age groups which were due to changes in hormonal levels, personal development, and the like (Kościński, 2011), but it did not track intraindividual variation in preferences in a longitudinal fashion. Kościński (2010) tested individual consistency of facial attractiveness assessment and found that self-correlation of women’s assessment was approximately 0.78, which means that about 40% (1–0.78^2^) of individual variation in attractiveness rating varies over time. To sum up, existing evidence suggests that preferences can change over time with age and reproductive stage of life, and that they can change in reaction to current circumstances (Rosenthal, 2017).

In short, it has been established that over time, mate preferences are to some degree plastic, but research of intraindividual stability in real mate choice in humans is sparse. To the best of our knowledge, only three studies so far tested individual consistency of mate choice (Eastwick et al., 2017; Newman et al., 2018; Štěrbová et al., 2018). They found consistency in preferences for eye color (Štěrbová et al., 2018; but cf. Newman et al., 2018), hair color (Štěrbová et al., 2018), attractiveness, masculinity, vitality, depression, delinquency, religiosity, educational aspirations, self-esteem, and intelligence (Eastwick et al., 2017). It is important to note, however, that the effect sizes were rather low. Nevertheless, it can be assumed that mate choice is affected by a vast array of demographic, physical, and personality traits.

In the present study, we tested individual consistency of mate choice with respect to traits that play an important role in human mating context and could therefore have a substantial impact on reproduction (Little, 2015). To wit, it is possible that different characteristics are valued in non-reproductive as opposed to reproductive relationships, that is, that different characteristics result in direct versus indirect benefits to offspring (Boothroyd and Vukovic, 2018).

The following is a list of characteristics we followed with respect to stability of individual mate preference in women:

### Tallness

Existing research suggests that women tend to choose partners who are tall (Hensley, 1994) and, in particular, taller than themselves (see meta-analysis, Pierce, 1996). This may be due to a link between body height and health (Christensen et al., 2007), and/or height and career success (Judge and Cable, 2004).

### Body Form

Preferences for body shape and weight may reflect environmental variation in food availability (Anderson et al., 1992), but also serve as cues to an individual’s social and economic status (Sarlio-Lahteenkorva and Lahelma, 1999). In general, optimal body mass index is perceived as attractive (Tovée et al., 1999). On top of that, metabolically expensive physical features, such as muscularity, are supposed to be attractive to females because they advertise that energy gathered from the environment could be converted to reproduction-related activities (Kaplan and Gangestad, 2004). Some studies found that women prefer muscular, but not too muscular, men (Dixson et al., 2003; Frederick and Haselton, 2007). In general, research supports the inverted-U hypothesis of masculine traits (Frederick and Haselton, 2007). These ambiguous results could be explained by personality characteristics associated with masculinity, such as higher dominance but also lower honesty, cooperativeness, emotionality, and parental qualities (Perrett et al., 1998; Boothroyd et al., 2008). Some studies thus found female preference for masculinity (Cunningham et al., 1990), whereas other research found preference for femininity in males (Perrett et al., 1998). Similarly, both hirsuteness and beardedness are sexual dimorphic traits. As in masculinity, evidence regarding female preferences is mixed (see for review, Dixson and Rantala, 2015), which could be due to association between beards and body hair on the one hand and perceived dominance and aggressiveness on the other hand (Puts, 2010).

### Eye and Hair Color

Research shows that eye and hair color play an important role in some human populations (Frost, 2006; White and Rabago-Smith, 2011) because they can affect perceived trustworthiness (Kleisner et al., 2013), dominance (Kleisner et al., 2010), attractiveness (Laeng et al., 2006), and health status (Frost et al., 2017).

### Personality

Last but not least, it has been established that cross-culturally, some personality traits likewise play an important role in mate choice (Buss and Barnes, 1986). It has been shown that both men and women desire partners who score high on Agreeableness, Openness (Botwin et al., 1997), and Emotional Stability (Conroy-Beam et al., 2015). These characteristics contribute to cooperation and altruism (Jensen-Campbell and Graziano, 2001), and thereby have a positive impact on the couple’s reproductive success (Buss, 1991).

The main aim of our study was to examine individual consistency of mate choice in women. Specifically, we tested whether women repeatedly choose long-term partners with particular demographic, physical, and personality characteristics. In short, we tested intraindividual variability of female mate choice. Consistency of mate choice was measured by several methods (by consistency index, percentage of variance in partners’ trait values accounted for by the respondent, and by correlations). Effect sizes were estimated by stepwise randomization effect size assessment and stepwise estimation of shared effect size. Only mothers were recruited for the study because from an evolutionary perspective, the most important partner is the father of a woman’s child or children. We have therefore tested mutual similarity among all of women’s long-term (ex-)partners and tried to find whether the partner with whom they had a child or children is different from those partners with whom they did not reproduce.

## Materials and Methods

All procedures followed were in accordance with ethical standards of the relevant committee on human experimentation and with the Helsinki Declaration. The study was approved by the Institutional Review Board of Charles University, Faculty of Sciences (Approval number 2016/23). All participants were informed about the goals of the study and approved their participation by clicking button ‘I agree’ below the informed consent. Written informed consent was not obtained because the study was conducted online.

### Participants

#### Respondents

Respondents were recruited via social sites, such as Instagram and Facebook, and websites aimed at mothering, e.g., babyweb.cz and emimino.cz, via flyers distributed to gynecology offices and dormitories, and by emails sent to respondents from our earlier studies. The initial sample consisted of 1,331 individuals. We analyzed only data from women who met the following criteria: (i) age between 18 and 45 years, (ii) heterosexual orientation (Kinsey scale < 3), (iii) had at least two long-term partners (defined as committed relationship that is believed to have future prospects), (iv) shared household with their biological father until at least 12 years old (this study is part of a larger research aimed at the imprinting-like effect).

The final sample consisted of 537 respondents (mean age = 29.14, *SD* = 6.281, median = 29, min = 19, max = 45). Information provided by each of these respondents was used in at least one analysis. All respondents together had a total of 1,599 partners (partners per respondent: mean = 2.98, *SD* = 1.32, median = 3, min = 2, max = 10). The mean length of relationship was 5.07 years (*SD* = 4.99, median = 3.17, min = 0, max = 27). When fathers and non-fathers were analyzed separately, mean length of relationship between these two categories of partners differed (mean length of relationship with fathers in years = 8.44, *SD* = 5.65, median = 7.416, min = 0.25, max = 27, mean length of relationship with non-fathers in years = 2.61, *SD* = 2.32, median = 2, min = 0, max = 20).

In many cases, respondents did not report all 21 characteristics about all partners, which prevented us from including all respondents and all partners in all tests of mate choice consistency. Respective sample sizes did not differ substantially (the number of respondents: mean = 482.8, *SD* = 21.9, median = 481, min = 435, max = 516; Number of partners with known information: mean = 1.388, *SD* = 64.3, median = 1.376, min = 1.236, max = 1.491) and are all reported in the Appendix.

#### Measurements

Respondents reported a total of 21 characteristics (3 demographic and 13 physical characteristics, and 5 personality traits). Since some of these characteristics can change within a short period of time (e.g., beardedness), respondents were asked to indicate characteristics as they remember them from the time when the relationship was established.

Of demographic characteristics, they were asked to report the size of their partners’ and fathers’ residence (1 – metropolis, 2 – city, 3, town, 4 – village), education level (1 – elementary school, 2 – high school, 3 – college, 4 – university), and age difference between themselves and their long-term partners (in months; negative numbers indicate that a woman is older than her partner).

Physical characteristics were reported by selecting the relevant image stimuli of eye color (gray, blue, green, brown, and black), hair color (9 shades varying from light blond to black), facial masculinity (five images varying from low to high masculinity) (Johnston, 2006), beardedness (four images varying from clean shaven to fully bearded) (Dixson and Brooks, 2013), muscularity (six images varying from low to high muscularity) (Frederick and Haselton, 2007), relative height (six images varying from man-taller pattern to women-taller pattern) (Pawlowski, 2003), body mass index (six images varying from low to high BMI) (Allen et al., 2003), hirsuteness (five images varying from a low to a high level of hirsuteness) (Dixson et al., 2010), leg to body ratio (LBR) (five images varying from relatively short to long legs) (Swami et al., 2006). Further, respondents indicated their partners’ body weight (in kilograms), body height (in centimeters), and overall masculinity and attractiveness (using a 7-point verbally anchored Likert scale, ranging from ‘under average’ to ‘above average’).

To assess personality characteristics, we used the Ten-Item Personality Inventory (TIPI) (Gosling et al., 2003), which maps five domains: Emotional Stability, Extraversion, Openness, Agreeableness, and Conscientiousness. Responses for each item were indicated on a 7-point Likert scale ranging from ‘strongly disagree’ to ‘strongly agree.’ We used a method of translation and back-translation into the Czech language.

## Procedure

The test was administered online using the Qualtrics platform. At the outset, respondents were asked to confirm their informed consent. In order to examine consistency of mate choice, respondents described their all (ex-)partners using image stimuli to assess their physical traits, indicated their demographic characteristics, and answered questions in a personality assessment questionnaire. They indicated only those characteristics they clearly remembered, otherwise they were asked to skip the question. A total of 206 (38.36%) out of 537 individuals involved in the analysis failed to indicate at least one of their partners’ characteristics. To make the process easier on the participants, they indicated the names or assigned nicknames to all (ex-)partners at the beginning of questionnaire. These (nick)names were then displayed with each question, meaning the respondent did not have to remember the order of (ex-)partners and we could be reasonably certain that for each partner, we know all the characteristics the participant remembers. This study was part of a wider research, which is why respondents also answered questions pertaining to their fathers. The whole procedure took approximately 80 min.

## Analyses

All analyses described below were conducted using the R 3.5.1 software. The code is available at: https://github.com/costlysignalling/Mate-choice-consistency-2.

### Consistency Evaluation

Average difference between respondents’ partners (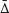) served as a measure of mate choice consistency. Larger differences between respondents’ partners indicate a more diverse set of partners and a lower mate choice consistency.

To assess the consistency of mate choice, we used a procedure similar to consistency index described in an earlier study (Štěrbová et al., 2018). Since the original consistency index views qualitative character states only in terms of identity (1) or difference (0) between pairs of respondent’s partners with respect to a particular character state, we used the average difference between respondents’ partners (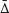) as a parametric equivalent of consistency index.

First, we assessed the average difference between a pair of partners separately for each respondent. For example, if only two long-term partners were reported, one had extroversion value 11 and the other 13, the average difference between them was 2 (i.e., 13 – 11). When four long-term partners were reported, their extroversion values could be as diverse as 5, 10, 7, and 14. In such a case, we calculated mutual differences for every possible pair of partners (10 – 5 = 5, 7 – 5 = 2, 14 – 5 = 9, 10 – 7 = 3, 14 – 10 = 4, 14 – 7 = 7) and then computed the average, i.e., (5 + 2 + 9 + 3 + 4 + 7)/6 = 5. This average value characterizes a woman’s mate choice consistency with respect to a particular trait. These individual values were later averaged across all respondents in the sample to evaluate overall mate choice consistency [in this short example, that value would be calculated as (2 + 5)/2 = 3.5]. This way, we ensured that every woman contributed to population consistency equally, i.e., regardless of the number of her long-term partners.

Populational average difference between all partners of an individual could thus be expressed as:

$$\bar{\Delta }=\sum_{i=1}^{n}\sum_{j=1}^{p_{i}-1}\sum_{k=j+1}^{p_{i}}\frac{\left|t_{ij}-t_{ik}\right|}{(p_{i}-1)/2n}$$

where 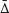 indicates the populational average difference between partners of an individual, p_i_ the number of partners of *i*-th individual, t_ix_ trait value of *x*-th partner of *i*-th individual, and *n* the number of respondents.

### Permutation Test of Mate Choice Consistency

Subsequently, we compared the observed average difference between an individual’s partners (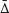) with the distribution of expected 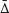 in a population with random pairing. A permutation test was executed to obtain the equivalent of a unidirectional test *p*-value.

We assigned partners to respondents randomly while maintaining the number of partners each respondent actually reported. This was done for each trait separately. We generated 10,000 such random populations and calculated the 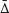 for each one. This yielded the distribution of 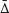 for a random pairing.

We assessed the proportion of 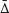 in random permutations which were smaller than the observed value of 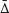. This gave us the equivalent of one-tailed test *p*-value, which indicated whether people were indeed significantly more consistent in their mate choice than one would expect if the choice were random.

### Stepwise Randomization Effect Size Assessment

This procedure allowed us to estimate the proportion of partners that have to be switched between respondents in order to lower the mate choice consistency to the expected level. This measure can range between 0% (observed consistency is lower than or equal to the expected consistency and no partners need to be switched) and 50% (see the example below).

We calculated the effect size attributable to consistency of mate choice using a stepwise randomization test. In this test, the observed 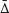 is gradually elevated by random relocation of one partner at a time until the expected value of 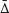 is reached (the procedure is described in detail in Štěrbová et al., 2018). The resulting percentage indicates the proportion of partners that needs to be switched among participants until one arrives at a 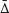 that would be expected in a random pairing. This was done 10,000 times for each trait. The mean value is reported as the estimated effect size together with a 95% confidence interval of this measure calculated as a 2.5–97.5% quantile of these 10,000 permutation-yielded percentages. It should be noted that though expressed as a percentage, this effect size is not identical to the proportion of explained variance. Maximal effect size in terms of percentage of partners that need to be relocated is about 50% because after relocating one half of all partners, one necessarily starts approaching the initial configuration again.

For instance, imagine a hypothetical dataset where every respondent reports 2 partners who have the same, either brown or blue, eye color. The observed 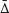 in such a population is 0. The maximal 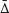 is observed when data are permuted so that each individual has one brown-eyed and one blue-eyed partner. This state is achieved when 50% of partners switch places. Relocation of one more blue-eyed or black-eyed partner would necessary result in coupling with another partner of the same eye color, which would lower the overall 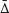.

### Other Measures of Mate Choice Consistency

Several non-permutation methods based on correlational approach had been proposed to tackle the mate choice consistency problem. These methods are to some extent equivalent to the proportion of partners to be switched between respondents (see section “Stepwise Randomization Effect Size Assessment”), but they are expressed either as a correlation coefficient or as the proportion of explained variance (values between 0 and 1 or 0 and 100%, respectively).

As suggested earlier (Eastwick et al., 2017), the percentage of variance in partners’ trait values accounted for by the respondent (i.e., the metric conceptually identical to the Intraclass correlation coefficient) can be used as a measure of mate choice consistency in parametric variables. We calculated this measure as well to enable a comparison with stepwise randomization effect size. We treated respondent identity (ID) as a random factor in a mixed-effect model (using lmer function within the lmerTest package). The statistic of interest was the random variance estimate for respondent ID divided by total variance. Reasonable benchmarks of this variance estimate were outlined at 10% for meaningful, 20% for a medium-sized effect, and 30% for a large effect (Kenny et al., 2006).

We have also calculated a simple Pearson correlation coefficient between two vectors of partners’ trait values. Every possible pair of partners of the same individual was treated as a unit of analysis. Individuals who had more partners therefore contributed to the overall coefficient disproportionally. Comparisons between this measure and more rigorously estimated effect sizes described above might indicate, however, that this is not necessarily a problem.

Pearson correlation coefficients between these three effect size measurements were calculated to demonstrate the equivalence of these measures. Additionally, we evaluated a linear model of dependence between the explained variance and the proportion of partners that needs to be switched between partners. This provided a useful tool for future comparisons with results on mate choice consistency that would use different approaches to effect size reporting.

### Stepwise Estimation of Shared Effect Size

Elaborating on the permutational effect size estimation (see section “Stepwise Randomization Effect Size Assessment”), we can assess shared effect size between mate choice consistency along two non-independent variables. Permutational effect size is expressed in the proportion of partners that needs to be switched between respondents. Shared effect size is the proportion of partners switched in two seemingly independent estimates of consistency effect size where correlation between variables is taken into account. If one switches 16% of partners to reach the expected consistency in body weight, and then another 6% are switched to avoid also consistency in body height, one could claim that 22% of all partners need to be switched to avoid non-random consistency in both height and weight. Going in the opposite direction, we relocate 11% to avoid consistency in height and then another 11% to avoid consistency in weight. Since the sum of residual effect sizes (6% + 11% = 17%) is lower than the sum of simple effect sizes (16% + 11% = 27%), one can assume non-independence between these variables and calculate a shared effect size. This ‘overlap’ is missing in the sum of residual effect sizes 22–17% and present twice in the sum of simple effect sizes 27–22%, but in both cases, the resulting proportion is 5%. These shared effect sizes can be used to calculate the maximal cumulative effect size, i.e., the number of partners that need to be switched between individuals to avoid mate choice consistency on all characteristics.

The link between every pair of partners’ qualities was assessed in two ways, namely Pearson correlation coefficient with a single partner as a unit of analysis and shared effect size, which is equivalent to the abovementioned stepwise randomization effect size for a pair of variables (A and B). Here, the 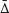 in variable A of empirical population is elevated by a stepwise reassignment of partners until the mean expected value of consistency with respect to A is reached. This rearranged population is then taken as a starting point and stepwise randomization effect size assessment is executed for variable B. The residual proportion of partners that need to be switched to avoid consistency in B is estimated after the effect of consistent mate choice with respect to A is eliminated. This is done 1,000 times to get the average residual effect size of B, and 1,000 times in the opposite direction to get the equivalent measure for A. Shared effect size is then calculated easily as *A + B* = (*A* ∩¬*B*) + (*B* ∩¬*A*) + 2 × (*A* ∩ *B*), where ∩ represents intersection, ∪ unity, and ¬ a set complement. This value was calculated for every pair of assessed variables.

Maximal cumulative effect size was then derived from pairwise shared effect sizes. Higher-order intersections were not estimated with permutation approach. It would have been possible, but extremely demanding with respect to computation time. Instead, we assumed that these intersections are proportional to the ratio of pairwise intersections. For example, if variables A, B, and C have effect sizes of 20, 15, and 10% partners to switch, and their shared effects are 10% for *A* ∩ *B*, 4% for *A* ∩ *C*, and 3% for *B* ∩ *C*, it is assumed that segments (*A* ∩ *C*) ∩¬*B*, (*B* ∩ *C*) ∩¬*A*, and *A* ∩ *C* ∩ *B* are in the same proportion as *A* ∩¬*B*, *B* ∩¬*A*, and *A* ∩ *B* (i.e., 10:5:10), and given that in the sum of *A* ∩ *C* and *A* ∩ *C* (4 + 3 = 7), segment *A* ∩ *B* ∩ *C* appears twice, it follows that the final proportions will be 2% for (*A* ∩ *C*) ∩¬*B*, 1% for (*B* ∩ *C*) ∩¬*A*, and 2% for *A* ∩ *B* ∩ *C*. The unique contribution of C [*C* ∩¬(*A* ∪ *B*)] must equal 5% of partners and the total cumulative effect size (*A* ∪ *B* ∪ *C*) must be 30%. To minimize possible errors stemming from inaccuracy of the assumption of intersection proportionality, variables were added to the total cumulative effect size one at a time according to a criterion of maximal unique contribution to the total effect size. First, we included variable A, which had the largest unique effect size, then we calculated for all other variables their unique contribution to the total effect size, selected the one which contributed the most, labeled it B, and included it in our calculation. For the next variable, we calculated its contribution to the union of A and B, added to the model the one with the largest unique contribution, and so on.

Growth of the unique contribution relative to the previous step of variable inclusion was a sign of accumulated error caused by inaccuracy of the assumption of intersection proportionality (i.e., in this step, the variable was rearranged back to high mate choice consistency). In each step, therefore, higher unique contributions were replaced by minimum values from contributions calculated in previous steps. This number represents the minimal possible contribution without allowing for a negative relationship between consistent mate choice along different variables. As a result, consistent mate choice in one variable or a union of variables could lead to inconsistent mate choice in another variable or variables. Maximal cumulative effect size was calculated as the total sum after the stepwise addition of all variables. The fact that a negative relationship between consistency on different variables was neglected is not problematic because the individual contributions still add up to the same total. Sacrifice of a consistent mate choice on one variable is compensated by an equivalent increase in the consistency along other variables. Therefore, although the order of unique contributions to overall consistency and their magnitude may be burdened by an error, the estimate of maximal cumulative effect size is sound and reliable.

Since a high number of assessed mate choice consistencies and their shared effects naturally leads to a substantial maximal cumulative effect size, the empirical level is contextualized with the expected maximal cumulative effect size in a population with random pairing. In this resampled population, the identity of partners and links between their qualities remained identical to the empirical data, but partners were scrambled among respondents so the total number of partners any respondent had remained unchanged.

### Differences Between Fathers and Non-fathers

Differences between partners with whom the respondents had children (‘fathers’) and other former long-term partners (‘non-fathers’) were investigated along all 21 romantic partner qualities our study had followed. Mean values and variances of fathers and non-fathers were compared to reveal possible differences between these groups. Changes in 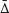 after the exclusion of fathers were compared to expected changes in 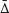 after the exclusion of random individuals to assess whether fathers were especially typical of given partner sets and elevated overall mate choice consistency, or exceptional within these sets, thus lowering overall mate choice consistency.

The 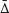 was calculated first for the full partner set and then for a restricted sample where fathers were excluded. The observed difference between these two samples was compared with the distribution of expected differences yielded by a permutation test. In each referential permutation, fathers were selected randomly from sets of partners provided by respondents. For instance, if a participant reported four long-term partners and two of them fathered at least one of her children, two individuals from this set were labeled as fathers and excluded in each permutation run (as expected, however, most respondents had children with only one partner). Two tailed *p*-value was calculated as a measure of significance of the difference between measured and expected changes in mate choice consistency after the exclusion of fathers. 10,000 permutation runs were executed for each variable. Where non-father 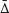 was significantly higher than expected, fathers were highly typical (or intermediate) representatives of woman’s partners. Where it was lower than expected, fathers were rather exceptional individuals within the sets of partners and measured consistency of mate choice was higher without them.

Yet even if fathers fitted in partner sets without being either exceptionally typical or highly atypical, it was still possible that there are some differences between fathers and non-fathers along the assessed qualities. Mixed effect models were employed to calculate the probability of equality of group means. Mixed effect equivalents of Levene’s test, where distance from the group mean is used as a response variable, were then used to investigate equality of variances between father and non-father groups, since it could be the case that even if the two groups do not differ in their means, the extreme or intermediate individuals just may not be the right ‘father material.’ We treated respondent ID as a random factor in all mixed effect models and used the lmer function lmer from the lmerTest package.

All independent sets of *p*-values reported in the result section were adjusted for multiple comparisons using the Benjamini–Hochberg procedure. Vectors of *p*-values calculated for the sets of 21 qualities were adjusted separately, while the *p*-values of correlations between qualities were adjusted together.

## Results

**FIGURE 1:**
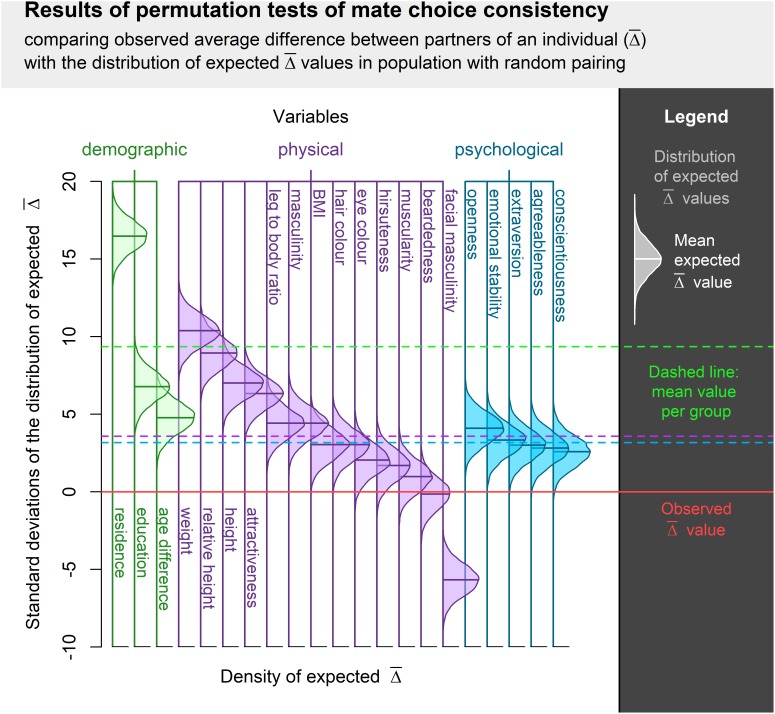
Visualization of permutation tests of mate choice consistency centered around observed 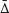 and normalized along the SD of expected 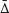 distribution. Difference between the observed and expected value is expressed in standard deviations from the expected value distribution. The higher the bell curve above the Observed 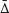 value, the higher the actual mate choice consistency. Bell curve below Observed 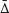 value indicates a trait where the observed mate choice was less consistent than expected.

Mate choice consistency was higher than expected in all assessed qualities except for facial masculinity and beardedness. Difference between observed and expected consistency was statistically significant in most qualities, but effect sizes differed substantially. While consistency of mate choice in residence or weight was substantial, it was only medium-sized or small with respect to hair or eye color. Complete results are summarized in Table 1 and Figure 1.

**Table 1:** Mate choice consistency: complete results.

	Observed 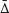	Expected 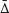	*SD* (of expected 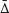 )	*p*-value	Proportion of partners to switch (%) (95% CI)	Respondent variance (%)	Pearson *r*
Residence	0.72	1.23	0.03	<0.001	19.48(18.03,20.9)	43.58	0.42
Education	0.72	0.87	0.02	<0.001	7.17(6.45,7.88)	19.24	0.18
Weight	8.82	11.31	0.24	<0.001	16.21(14.04,18.39)	33.77	0.31
Height	6.3	7.42	0.16	<0.001	11.28(9.53,13.06)	24.56	0.22
Age difference	44.05	49.44	1.13	<0.001	10.49(8.41,12.7)	13.97	0.14
Attractiveness	1.23	1.44	0.03	<0.001	8.28(7.26,9.36)	16.49	0.19
Masculinity	1.26	1.4	0.03	<0.001	5.51(4.61,6.44)	9.71	0.11
Eye color	1.28	1.35	0.03	0.026	2.76(2.17,3.43)	4.31	0.05
Hair color	2.54	2.74	0.07	0.001	4.81(3.89,5.77)	10.17	0.12
Facial masculinity	1.66	1.46	0.04	1.000	0.07(0.07,0.07)	0	-0.05
Beardedness	0.67	0.67	0.02	0.575	0.07(0.07,0.07)	6.83	0.08
Muscularity	1.01	1.03	0.03	0.181	1.27(0.87,1.67)	3.43	0.05
BMI	1.25	1.35	0.03	0.002	4.23(3.5,4.99)	11.87	0.17
Relative height	1.18	1.5	0.04	<0.001	11.99(10.75,13.3)	30.58	0.34
Hirsuteness	1.41	1.48	0.04	0.055	2.56(1.93,3.26)	10.31	0.12
Leg to body ratio	1.21	1.35	0.03	<0.001	5.93(4.98,6.89)	13.52	0.12
Extraversion	3.12	3.36	0.08	0.002	5.25(4.2,6.41)	9.32	0.12
Agreeableness	2.94	3.14	0.07	0.003	5.07(4.1,6.11)	3.55	0.06
Conscientiousness	3.68	3.92	0.09	0.006	4.68(3.62,5.76)	5.08	0.09
Emotional stability	3.29	3.57	0.08	0.001	5.84(4.69,7.08)	8.1	0.12
Openness	2.91	3.21	0.07	<0.001	6.94(5.77,8.16)	9.27	0.11

The average effect size was highest in demographic variables, but none of the pairwise comparisons between groups of variables (demographic, physical, and psychological) was statistically significant (*p* > 0.1). Permutation test results are visualized in Figure 1. All sample sizes and descriptive statistics of all variables are listed in the Appendix. The different estimates of effect size were highly correlated. The proportion of males who had to be relocated between respondents correlated with the variance accounted for by the respondent at 0.93, whereby a linear model of relationship between these two measures supports the idea that the latter is approximately double of the former. The slope in the model where respondent-attributable variance regressed on the proportion of partners to relocate was 2.08 (95% CI = 1.72–2.45) with minimal (not significantly different from 0) intercept of -0.18 (95% CI = -3.19–2.83). Results yielded by the simple Pearson correlation correlated at 0.91 with the percentage of partners to relocate and at 0.98 with respondent-attributable variance. All of these measures can be thus treated as functionally equivalent.

**FIGURE 2:**
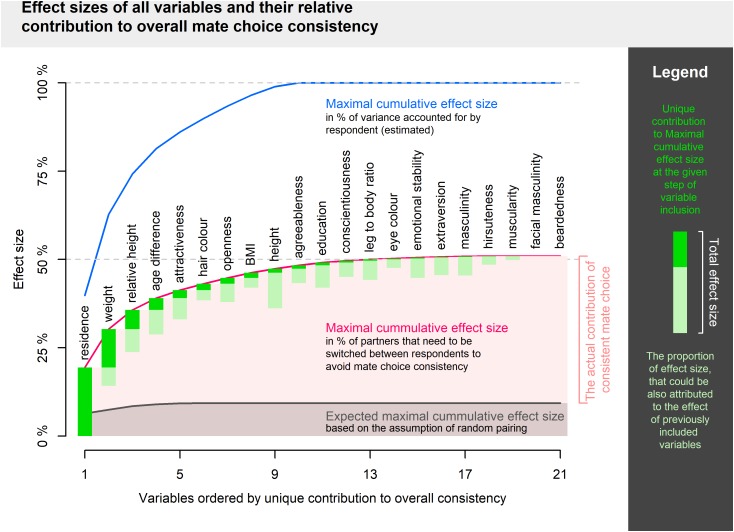
Visualization of maximal cumulative effect size. Variables are added in order given by maximal unique contribution to overall consistency.

Links between pairs of partners’ qualities are summarized in Table 2. In total, 103 out of 210 correlations were significant even after Benjamini–Hochberg correction for multiple comparisons. Maximal cumulative effect size was 50.95% (expressed in the proportion of partners to switch between individuals). The first 10 variables ordered according to their unique contribution starting with the highest (residence, weight, relative height, age difference, attractiveness, hair color, openness, BMI, height, agreeableness, in this order) explained 48.30% of partner assignment. The other 11 variables contributed little (their unique contributions were less than 1%) or not at all (after the inclusion of all other variables, facial masculinity and beardedness failed to show any positive numbers). Full results are visualized in Figure 2.

**Table 2:** Relations between investigated qualities of romantic partners expressed in shared effect sizes and Pearson correlations.

	Residence	Education	Weight	Height	Age difference	Attractiveness	Masculinity	Eye Color	Hair Color	Facial masculinity	Bearded ness	Muscularity	BMI	Relative height	Hirsuteness	Leg to body ratio	Extraversion	Agreeableness	Conscientiousness	Emotional stability	Openness
Residence	19.4%	-0.26^∗^	-0.02	-0.06^∗^	-0.01	-0.03	-0.04	-0.07^∗^	-0.08^∗^	-0.04	-0.04	-0.03	-0.01	-0.02	0.02	-0.07^∗^	-0.01	0.03	0.00	0.01	-0.11^∗^
Education	3.2%	7.1%	0.06	0.12^∗^	-0.07^∗^	0.04	0.05	0.00	0.04	0.01	0.02	0.03	0.04	-0.03	0.09^∗^	0.10^∗^	-0.07^∗^	0.13^∗^	0.27^∗^	0.16^∗^	0.10^∗^
Weight	5.3%	1.8%	16.1%	0.48^∗^	0.17^∗^	-0.02	0.26^∗^	-0.01	0.05	0.09^∗^	0.16^∗^	0.24^∗^	0.59^∗^	-0.25^∗^	0.27^∗^	0.02	0.04	-0.02	0.08^∗^	0.05	0.03
Height	4.0%	1.6%	5.0%	11.2%	0.01	0.08^∗^	0.14^∗^	-0.03	0.02	0.19^∗^	0.05	0.07^∗^	0.04	-0.58^∗^	0.03	0.44^∗^	0.03	0.03	0.06	0.09^∗^	0.04
Age difference	3.5%	1.5%	3.3%	2.5%	10.3%	-0.03	0.13^∗^	-0.01	0.08^∗^	0.15^∗^	0.14^∗^	0.07^∗^	0.18^∗^	0.06	0.14^∗^	-0.04	0.05	-0.03	0.03	-0.01	-0.01
Attractiveness	2.7%	1.4%	2.5%	2.1%	1.7%	8.3%	0.45^∗^	0.02	0.09^∗^	0.19^∗^	0.09^∗^	0.28^∗^	-0.13^∗^	-0.05	0.00	0.07^∗^	0.19^∗^	0.17^∗^	0.10^∗^	0.18^∗^	0.19^∗^
Masculinity	1.8%	0.8%	2.2%	1.3%	1.4%	2.1%	5.4%	0.01	0.05	0.28^∗^	0.16^∗^	0.50^∗^	0.18^∗^	-0.10^∗^	0.18^∗^	-0.04	0.16^∗^	0.11^∗^	0.13^∗^	0.19^∗^	0.18^∗^
Eye color	1.1%	0.3%	0.7%	0.6%	0.6%	0.4%	0.2%	2.7%	0.39^∗^	0.03	0.04	-0.03	-0.02	-0.01	0.04	-0.03	0.03	0.00	-0.07^∗^	-0.03	0.00
Hair color	1.2%	0.7%	1.0%	0.9%	0.3%	0.7%	0.2%	0.6%	4.7%	0.08^∗^	0.15^∗^	0.03	0.02	0.01	0.17^∗^	0.01	0.02	-0.01	-0.03	-0.02	0.02
Facial masculinity	0.0%	0.0%	0.0%	0.0%	0.1%	0.0%	0.0%	0.0%	0.0%	0.0%	0.19^∗^	0.31^∗^	-0.08^∗^	-0.12^∗^	0.16^∗^	0.15^∗^	0.04	-0.02	0.00	0.06	0.06
Beardedness	0.0%	0.0%	0.0%	0.0%	0.0%	0.0%	0.0%	0.0%	0.0%	0.0%	0.0%	0.13^∗^	0.14^∗^	-0.03	0.34^∗^	-0.01	0.07^∗^	0.06	0.02	0.08^∗^	0.12^∗^
Muscularity	0.4%	0.1%	0.6%	0.2%	0.1%	0.5%	0.5%	0.1%	0.3%	0.0%	0.0%	1.2%	0.19^∗^	0.01	0.17^∗^	-0.08^∗^	0.09^∗^	0.10^∗^	0.12^∗^	0.15^∗^	0.13^∗^
BMI	0.6%	0.1%	1.7%	0.3%	0.2%	0.5%	0.6%	0.0%	0.1%	0.0%	0.0%	0.4%	4.2%	0.01	0.30^∗^	-0.25^∗^	0.02	-0.01	0.08^∗^	0.00	-0.01
Relative height	4.1%	1.4%	3.7%	4.9%	2.8%	2.3%	1.2%	0.6%	0.8%	0.0%	0.0%	0.3%	0.4%	11.9%	0.01	-0.39^∗^	0.00	-0.02	-0.05	-0.06	0.00
Hirsuteness	0.9%	0.2%	1.0%	0.3%	0.5%	0.4%	0.4%	0.0%	0.1%	0.0%	0.0%	0.2%	0.4%	0.4%	2.4%	-0.10^∗^	0.01	0.03	0.08^∗^	0.01	0.03
Leg to body ration	2.0%	0.7%	1.6%	2.6%	1.3%	1.1%	0.8%	0.1%	0.3%	0.0%	0.0%	0.2%	0.4%	2.4%	0.5%	5.8%	-0.05	0.02	0.03	0.04	0.02
Extraversion	1.9%	0.7%	1.7%	1.3%	0.9%	1.2%	0.5%	0.3%	0.5%	0.0%	0.0%	0.1%	0.2%	1.2%	0.4%	0.6%	5.1%	0.09^∗^	-0.09^∗^	0.17^∗^	0.38^∗^
Agreeableness	1.8%	0.5%	1.6%	0.7%	0.2%	0.9%	0.9%	0.1%	0.2%	0.0%	0.0%	0.2%	0.2%	0.5%	0.4%	0.5%	1.0%	5.0%	0.33^∗^	0.48^∗^	0.22^∗^
Conscientiousness	1.7%	0.7%	1.0%	0.8%	0.7%	0.9%	0.9%	0.1%	0.1%	0.0%	0.0%	0.0%	0.0%	0.6%	0.3%	0.3%	0.9%	1.2%	4.6%	0.38^∗^	0.08^∗^
Emotional stability	2.2%	0.9%	2.0%	1.4%	1.0%	1.1%	0.8%	0.2%	0.3%	0.0%	0.0%	0.3%	0.3%	0.9%	0.5%	0.5%	1.2%	2.0%	1.3%	5.8%	0.19^∗^
Openness	2.5%	1.0%	1.6%	1.2%	1.2%	1.3%	0.8%	0.2%	0.5%	0.0%	0.0%	0.3%	0.3%	1.4%	0.3%	0.4%	1.6%	1.2%	1.0%	1.5%	6.9%

*Indicated on the diagonal is the total effect size in terms of proportion of partners who have to be switched between respondents to avoid non-random consistency of mate choice along a given variable (see section “Stepwise Randomization Effect Size Assessment”). Shared effect sizes, i.e., proportions of partners that one switches in both seemingly independent estimations of consistency effect size due to correlation between variables (see section “Stepwise Estimation of Shared Effect Size”), are located below the diagonal. Correlations between variables with partner as a unit of analysis are displayed above the diagonal. P-values are adjusted for multiple comparisons with Benjamini–Hochberg correction procedure. Significance levels after this correction are indicated: ^∗^p < 0.05.*

Reaching maximal possible effect size suggests that adding yet other variables to a similar model of cumulative consistency would add little to our current sum. On the other hand, it is conceivable that one might select precisely those variables which are not intercorrelated and explain a majority of mate choice consistency in just a handful independent dimensions. In theory, complex interaction patterns may lead to an even higher cumulative effect size since 50% of partners to relocate as an effect size limit applies to a single variable with two levels and represents the difference between maximal and minimal consistency (i.e., not maximal and expected). The high proportion of significantly correlated pairs of variables (49%), does, however, fit well within the impression of a substantial redundancy in our model.

**FIGURE 3:**
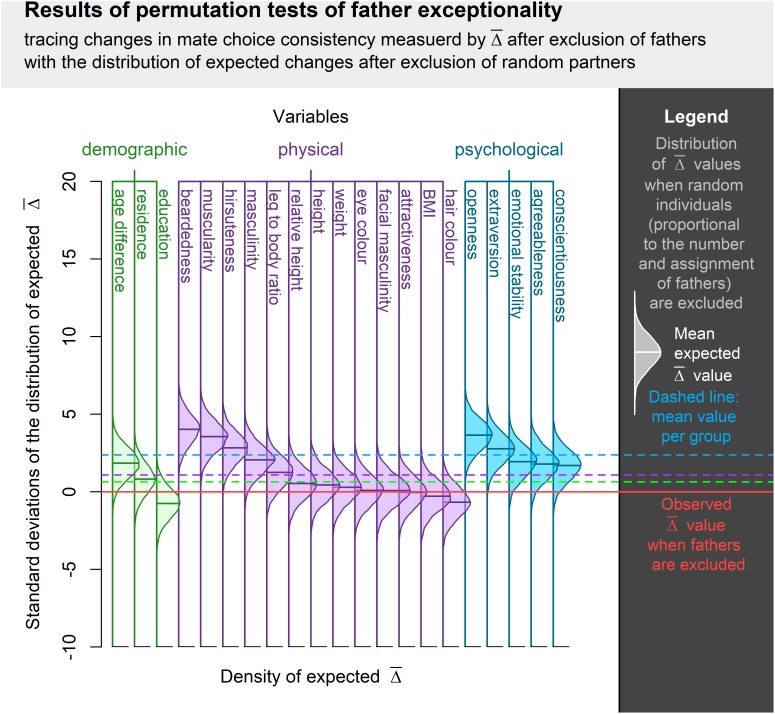
Visualization of permutation tests of father exceptionality centered around the observed 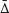 when fathers were excluded from the sample of partners and normalized along the SD of expected 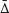 distribution in such a situation. Difference between observed and expected values is expressed in standard deviations of expected value distribution. The higher the bell curve above the observed 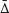 value, the more exceptional were the fathers among the long-term partners of an individual. Bell curve below the observed 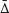 value indicates a trait where fathers were more typical representatives of an individual’s long-term partners.

Permutation test of changes in mate choice consistency revealed that fathers are significantly exceptional amongst participants’ long-term partners in beardedness, muscularity, hirsuteness, extraversion, and openness. The average 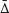 without these individuals was lower than the 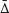 in permutation runs where an equivalent proportion of random partners (i.e., fathers and non-fathers) was excluded. Fathers were not significantly typical long-term partners in any of the assessed qualities. Complete results of these tests are summarized in Table 3 and visualization is provided in Figure 3.

**Table 3:** Permutation test of father exceptionality, complete results.

	Change in 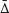 when fathers are excluded	Expected change in 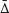	*SD* (of expected change in 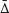 )	*p*-value
Residence	-0.01	0.02	0.03	0.717
Education	0.01	-0.01	0.03	0.725
Weight	0.08	0.16	0.28	0.925
Height	-0.18	-0.10	0.19	0.865
Age difference	-2.96	-0.32	1.42	0.176
Attractiveness	0.03	0.02	0.04	0.984
Masculinity	-0.07	0.01	0.04	0.141
Eye color	-0.01	-0.01	0.04	0.978
Hair color	-0.02	-0.08	0.08	0.755
Facial masculinity	-0.05	-0.05	0.04	0.978
Beardedness	-0.15	-0.04	0.03	0.002
Muscularity	-0.14	-0.03	0.03	0.002
BMI	0.04	0.03	0.04	0.925
Relative height	-0.01	0.01	0.04	0.834
Hirsuteness	-0.24	-0.11	0.05	0.024
Leg to body ratio	-0.08	-0.03	0.04	0.409
Extraversion	-0.29	-0.01	0.10	0.024
Agreeableness	-0.13	0.04	0.09	0.176
Conscientiousness	-0.24	-0.05	0.12	0.191
Emotional stability	-0.29	-0.09	0.10	0.158
Openness	-0.42	-0.08	0.09	0.004

In qualities where fathers were indicated as exceptional individuals (except for extraversion), mean trait values differed between fathers and non-fathers, while variances differed in beardedness, muscularity, and hirsuteness. Fathers were more bearded, hairier, more muscular, and showed a higher openness to experience. These differences might explain the overall exceptionality of fathers except for extraversion. It seems that fathers are outliers within partner sets even where the group means and variances of father and non-father sets do not differ. Moreover, fathers lived in larger cities, had higher education, were heavier and taller (although relatively, their height was closer to the height of respondents), more attractive and masculine, had lighter eyes, darker hair, more masculine faces, and were more agreeable, conscientious, and emotionally stable than non-fathers.

**FIGURE 4:**
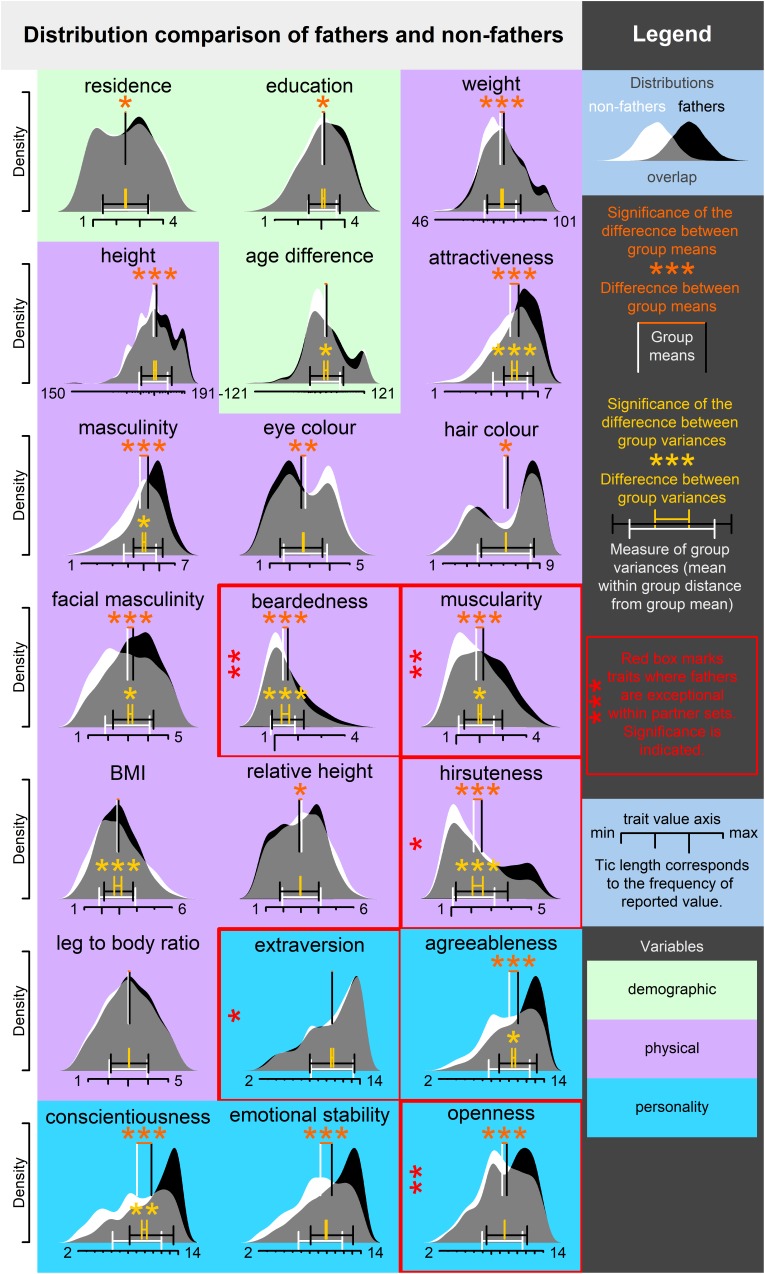
Visualization of differences between fathers and non-fathers. Significance of difference between group means and variances is estimated from mixed effect models with respondent ID treated as a random factor. Significance levels are indicated as follows: ^∗^*p* < 0.05, ^∗∗^*p* < 0.01, ^∗∗∗^*p* < 0.001.

Group variances differed in several qualities. Fathers were significantly more variable than non-fathers with respect to age difference from the respondent and less variable in attractiveness, masculinity (general and facial), BMI, conscientiousness, and agreeableness. It seems that along these variables, either or both of the extremes are not the right for the ‘father material’. A graphic overview which compares densities that indicate differences between group means and variances is presented in Figure 4. Complete results in a textual form are listed in Table 4.

**Table 4:** Results of Mixed effect models comparing father/non-father means and variances.

	Comparison of mean values				Comparison of variances (within group residuals as in Levene’s test)			
	Intercept non-father	Effect father	Standard error	*p*-value	Intercept non-father	Effect father	Standard error	*p*-value
Residence	2.48	-0.1	0.04	0.04	0.98	0	0.02	0.837
Education	3.03	0.1	0.04	0.015	0.61	0.05	0.03	0.064
Weight	78.18	1.98	0.45	<0.001	7.9	0.39	0.29	0.27
Height	179.66	1.19	0.31	<0.001	5.2	0.34	0.19	0.135
Age difference	34.52	3.67	2.31	0.132	32.97	3.99	1.47	0.02
Attractiveness	5.18	0.57	0.06	<0.001	1.1	-0.16	0.04	<0.001
Masculinity	4.73	0.55	0.06	<0.001	1.04	-0.1	0.04	0.025
Eye color	2.79	-0.21	0.06	0.001	1.08	-0.02	0.03	0.52
Hair color	6.05	0.3	0.12	0.018	2.19	-0.01	0.05	0.837
Facial masculinity	2.97	0.29	0.07	<0.001	1.11	-0.1	0.04	0.016
Beardedness	1.35	0.22	0.04	<0.001	0.52	0.17	0.02	<0.001
Muscularity	1.84	0.32	0.05	<0.001	0.75	0.06	0.03	0.046
BMI	2.88	0.07	0.06	0.238	1.02	-0.2	0.04	<0.001
Relative height	3.04	-0.14	0.06	0.025	1.08	0.03	0.04	0.52
Hirsuteness	2.12	0.41	0.07	<0.001	1.04	0.26	0.04	<0.001
Leg to body ratio	3	0.08	0.06	0.222	0.95	-0.01	0.04	0.837
Extraversion	10.67	0.01	0.16	0.971	2.5	0.17	0.08	0.086
Agreeableness	10.04	1	0.14	<0.001	2.32	-0.18	0.08	0.049
Conscientiousness	9.05	1.76	0.17	<0.001	2.94	-0.33	0.09	0.001
Emotional stability	9.22	1.39	0.16	<0.001	2.57	-0.08	0.09	0.514
Openness	9.23	0.56	0.14	<0.001	2.32	0.02	0.08	0.837

*Respondent ID is treated as a random factor.*

## Discussion

The aim of this study was to examine consistency of mate choice with respect to a variety of demographic, physical, and personality characteristics. We found that women choose long-term partners consistently across all types of characteristics (demographic, physical, and personality), but consistency was not observed in all tested traits. We also investigated potential differences in tested characteristics between long-term ex-partners and partner(s) with whom women had child(ren). Results revealed that fathers in general fit the women’s ‘type,’ although differences between them and other (ex-)partners are not large. Our findings are in line with earlier research (Eastwick et al., 2017; Štěrbová et al., 2018 but cf. Newman et al., 2018), which found that people consistently choose partners with certain traits, although reported effect sizes were rather small.

Is there any potential advantage to having a ‘type’? We could assume that preference for a particular ‘type’ may facilitate mate choice decisions. In theory, the pool of potential partners is immense and in the most extreme case covers almost one half of adult human population on Earth. This theoretical pool is, of course, unrealistic, but even so, people do have many potential partners to actually choose from. In order to navigate this vast amount of opportunities, it may be useful to follow a certain direction in this multidimensional trait space of human characteristics. Preference for a certain ‘type’ would constrain the spectrum of potential choices and reduce the dimensionality of trait space. A systematic, ‘type-directed’ exploration of this multidimensional trait space would facilitate better orientation on the ‘mating market.’ In short, having a ‘type’ means that women need not create new preferences always anew and based on random choices, i.e., it precludes them from jumping unsystematically across the vast dimensionality of trait space. A ‘type’ should not be viewed as a rigid attractor but rather as a polarizing filter which canalizes the selection of optimal partner. A ‘type’ is thus not a target in itself but rather the means by which a goal can be reached (and, e.g., an appropriate partner for reproduction, a father, found). This is why fathers do not fully correspond to a typical partner and show some, however, small, deviation from the type.

This setup of optimal partner preferences may be beneficial. Mate choice not guided by such relatively stable but to some degree flexible preferences would be much more cognitively demanding and time-consuming. What remains unclear, however, is when and how are these preferences established. One of such mechanisms could be the imprinting-like effect (parent–partner similarity) or homogamy (self-similarity) (see Štěrbová et al., 2018). Moreover, parent-partner similarity can be promoted by emotional closeness with a parent during childhood (Saxton, 2016). Some plasticity of preferences may be adaptive also because it helps individuals adjust their preferences according to the current situation (e.g., ecological circumstances, inner state, their own characteristics which vary over time, experiences). From an evolutionary perspective, variation in mate preferences is important for speciation and diversification (Rodríguez et al., 2013). Species can adapt to changing circumstances by adjusting their mate choice. One might assume that learning would decrease the consistency of mate choice, that one would, for instance, choose a partner with characteristics different from an earlier partner because of negative experiences. On the other hand, mate choice is a mostly non-conscious process, which implies that partner preferences are not easily modulated by experience. Our findings support these assumptions, because we found that women have a ‘type’ and choose partners who fit it.

One can only speculate whether mate choice when reproduction is in question differs from earlier preferences, i.e., preferences in a non-reproductive context. From an evolutionary perspective, the most important partner is the one with whom a woman will reproduce. This is why we tested whether fathers fit the women’s ‘type,’ or rather whether fathers’ characteristics differ from characteristics of the non-fathers.

Our results show that although consistency is found across all of woman’s long-term partners, there are some notable differences between non-fathers and fathers. In particular, fathers disrupted consistency in beardedness, hirsuteness, muscularity, extraversion, and openness. The means and variance differ significantly between fathers and non-fathers in many other characteristics as well. This could be due to several reasons. First of all, it is possible that men with whom women reproduce actually differ from those with whom they do not. It should be noted, however, that most characteristics vary over time. This finding may thus be a side effect of higher age of fathers compared to non-fathers, especially in those characteristics where fathers disrupt mate choice consistency. Secondly, differences between fathers and non-fathers might be due to time-dependent cultural shifts. For instance fashions concerning beardedness vary significantly over time, which may cause a higher mutual similarity among former partners (non-fathers) as opposed to fathers (the most recent partner). Moreover, from an evolutionary perspective, these slight differences among partners could be due to the fact that each partner could be a potential father of a woman’s children. It may be therefore beneficial for a woman not to experiment too much in her mate choice.

Nevertheless, some differences between fathers and non-fathers were observed. They could be due to individual relationship experience. In other words, it is possible that women adjust their mate choice depending on experiences gathered over lifetime and reproduce with a partner who fits their preferences better than earlier partners. Differences between fathers and non-fathers could also be due to memory bias or cognitive dissonance influenced by positive or negative experiences with particular partners. If so, the level of negative experiences with former partners should positively correlate with fathers’ non-typicality. Moreover, women might have a tendency to ascribe more positive characteristics to a current partner (usually the father of her child or children) than to their ex-partners. In other words, partnership status itself may have an impact on the assessment. Alternatively, former partners could be regarded on average more positively simply because women’s detailed memories of problems encountered in earlier relationships fade with time. There might be therefore some trade-offs between the principles of ‘my baby’s father is always better’ and ‘sweet recollections of past loves.’ We cannot address such possibilities in our analysis.

The fact that fathers lower the measured mate choice consistency and yet there is no meaningful systematic difference between fathers and non-fathers could be accounted for by either of two possible explanations. First of all, it is possible that women reproduce with men who have different characteristics than their ex-partners. This pattern was, however, found only for extroversion (whereby women who date extroverted men reproduced with more introverted individuals, while other women date introverts but reproduce with men who are more extroverted). Moreover, overall consistency of mate choice with respect to extroversion was high even when fathers were included in the partner sample and even in cases when fathers and non-fathers were excluded at random. It is then fair to assume that fathers do, after all, fit within the general type of women also in extraversion, although they tend to be on one of the extreme tails of this intrapersonal distribution. The second possible explanation is that variance in father and non-father group differs and fathers are a more variable group. We did not, however, encounter such a case in our dataset. If fathers had a higher variance than non-fathers, they would have to have also a higher average trait value. Where this was not the case (age difference), we found that father exclusion did significantly elevate mate choice consistency.

These findings are limited by including only women in reproductive age, because preferences and potentially also actual choices can change in connection with changes in hormonal levels (e.g., menopause) during women’s lives (Boothroyd and Vukovic, 2018). Female preferences are underpinned by a set of evolutionary adaptations (Kokko et al., 2003; Geary et al., 2004) and can change with age so as to reflect women’s different interests (Kościński, 2011). Similarly, the importance of particular physical and personality characteristics can vary during one’s life. To test intraindividual variability in mate choice, future studies should therefore investigate women’s preferences and actual choices during their lives from childhood to menopause. Another limitation of our study is given by the fact that only respondents but not their (ex-)partners participated in the study. Although it would be nearly impossible to recruit also all (ex-)partners, it ought to be taken into account that when a woman reports about all of her partners during one session, this may lead to bias in a direction of mutual similarity. Alternatively, assessment of partners’ characteristics could be biased by subsequent experiences, memories, or circumstances of a break-up of a relationship.

Our results support the hypothesis of consistency of mate choice with respect to a variety of characteristics, but further research is needed to confirm this effect through a longitudinal design. Secondly, consistency in mate choice should be investigated also in men and in a short-term mating context, where consistency of mate choice may be lower than in a long-term context (Štěrbová et al., 2018). Furthermore, future research should investigate interindividual differences in individual consistency (to find out which characteristics predict a consistent mate choice, the role of family members, etc.). In the light of all of the above, it would also be highly relevant to investigate to what degree are preferences inherited or learned. A twin study (Germine et al., 2015) has reported that face preferences seem to be mainly explained by environmental variation, but more research in this field is needed. And finally, research should focus not only on actual choices but also on partner preferences.

## Author Contributions

ZŠ developed the study concept and collected data. PT performed the data analysis. ZŠ, PT, and KK interpreted results. ZŠ, PT, and KK drafted the manuscript. All authors approved the final version of the manuscript for submission.

## Conflict of Interest Statement

The authors declare that the research was conducted in the absence of any commercial or financial relationships that could be construed as a potential conflict of interest.
