# Supplementary material for: She Always Steps in the Same River: Similarity Among Long-Term Partners in Their Demographic, Physical, and Personality Characteristics
Source: Front Psychol. 2019 Feb 5;10:52. doi: 10.3389/fpsyg.2019.00052 (PMC6371050; doi:10.3389/fpsyg.2019.00052)
Supplement: Supplementary file 1 [file Table_1.DOCX]

**Appendix**

**Sample size overview**

**Table A1: Summary of assessed romantic partner qualities and sample sizes used in various tests**

|  | mean | SD | range | N respondents | N partners | N fathers | N respondents reporting fathers | N respondents after excluding fathers | N partners after excluding fathers |
| --- | --- | --- | --- | --- | --- | --- | --- | --- | --- |
| residence | 2.36 | 1.1 | 1–4 | 512 | 1464 | 536 | 506 | 270 | 702 |
| education | 3.07 | 0.81 | 1–4 | 516 | 1473 | 541 | 510 | 270 | 703 |
| weight | 79.15 | 10.07 | 46–101 | 494 | 1403 | 519 | 489 | 255 | 662 |
| height | 180.06 | 6.55 | 152–191 | 500 | 1416 | 524 | 494 | 259 | 668 |
| age difference | 35.41 | 44.71 | -121–121 | 434 | 1236 | 455 | 428 | 225 | 588 |
| attractiveness | 5.42 | 1.31 | 1–7 | 508 | 1474 | 530 | 502 | 274 | 725 |
| masculinity | 4.96 | 1.29 | 1–7 | 509 | 1476 | 530 | 503 | 270 | 719 |
| eye color | 2.71 | 1.21 | 1–5 | 500 | 1429 | 522 | 495 | 261 | 682 |
| hair color | 6.19 | 2.44 | 1–9 | 511 | 1491 | 528 | 503 | 280 | 743 |
| facial masculinity | 3.08 | 1.29 | 1–5 | 478 | 1374 | 495 | 470 | 256 | 670 |
| beardedness | 1.45 | 0.73 | 1–4 | 481 | 1376 | 500 | 474 | 255 | 664 |
| muscularity | 1.99 | 0.95 | 1–4 | 481 | 1379 | 499 | 473 | 259 | 672 |
| BMI | 2.92 | 1.22 | 1–6 | 482 | 1402 | 500 | 476 | 268 | 701 |
| relative height | 2.98 | 1.34 | 1–6 | 472 | 1368 | 488 | 465 | 260 | 680 |
| hirsuteness | 2.28 | 1.37 | 1–5 | 470 | 1350 | 487 | 463 | 256 | 662 |
| leg to body ratio | 3.05 | 1.2 | 1–5 | 453 | 1306 | 471 | 447 | 248 | 643 |
| extraversion | 10.73 | 3.04 | 2–14 | 472 | 1358 | 483 | 459 | 254 | 670 |
| agreeableness | 10.36 | 2.8 | 2–14 | 467 | 1343 | 477 | 454 | 250 | 662 |
| conscientiousness | 9.69 | 3.47 | 2–14 | 468 | 1353 | 481 | 457 | 251 | 668 |
| stability | 9.74 | 3.15 | 2–14 | 466 | 1342 | 480 | 456 | 247 | 656 |
| openness | 9.51 | 2.83 | 2–14 | 465 | 1335 | 478 | 454 | 248 | 653 |

Lower numbers after the exclusion of fathers are due to the fact that some individuals no longer qualified as suitable for our study because after excluding one partner (father or nonfather), they had only one partner and $\bar{\Delta}$ therefore could not be calculated.
